# Cytotoxicity of Thioalkaloid-Enriched *Nuphar lutea* Extract and Purified 6,6′-Dihydroxythiobinupharidine in Acute Myeloid Leukemia Cells: The Role of Oxidative Stress and Intracellular Calcium

**DOI:** 10.3390/ph15040410

**Published:** 2022-03-28

**Authors:** Suchismita Muduli, Avi Golan-Goldhirsh, Jacob Gopas, Michael Danilenko

**Affiliations:** 1 Department of Clinical Biochemistry & Pharmacology, Faculty of Health Sciences, Ben-Gurion University of the Negev, Beer Sheva 8410501, Israel; muduli@post.bgu.ac.il; 2 The Jacob Blaustein Institutes for Desert Research (BIDR), French Associates Institute for Agriculture and Biotechnology of Drylands, Ben-Gurion University of the Negev, Sede Boqer Campus, Midreshet Ben Gurion 8499000, Israel; avigolan@bgu.ac.il; 3 Department of Microbiology, Immunology & Genetics, Faculty of Health Sciences, Ben-Gurion University of the Negev, Beer Sheva 8410501, Israel; jacob@bgu.ac.il; 4 Department of Oncology, Soroka University Medical Center, Beer Sheva 8410101, Israel

**Keywords:** acute myeloid leukemia (AML), water lily (*Nuphar lutea*) extract (NUP), 6,6′-dihydroxythiobinupharidine (DTBN), apoptosis, reactive oxygen species (ROS), oxidative stress, intracellular calcium

## Abstract

Acute myeloid leukemia (AML) is an aggressive hematological malignancy characterized by uncontrolled proliferation of immature myeloid progenitors. Here, we report the in vitro antileukemic effects of the sesquiterpene thioalkaloid-enriched fraction of the *Nuphar lutea* leaf extract (NUP) and a purified thioalkaloid 6,6′-dihydroxythiobinupharidine (DTBN). Treatment with 0.3–10 µg/mL NUP caused a dose- and time-dependent reduction in proliferation and viability of human AML cells (KG-1a, HL60 and U937). This was associated with apoptosis induction manifested by annexin-V/propidium iodide binding as well as cleavage of caspases 8, 9, and 3 as well as poly (ADP-ribose) polymerase. Caspase-dependence of the apoptotic effect was confirmed using the pan-caspase inhibitor Q-VD-OPH. NUP induced significant biphasic changes in the cytosolic levels of reactive oxygen species (ROS) compared to untreated cells—a decrease at early time points (2–4 h) and an increase after a longer incubation (24 h). ROS accumulation was accompanied by lowering the cellular glutathione (GSH) levels. In addition, NUP treatment resulted in elevation of the cytosolic Ca^2+^ (Ca^2+^_cyt_) levels. The thiol antioxidant and glutathione precursor N-acetyl cysteine prevented NUP-induced ROS accumulation and markedly inhibited apoptosis. A similar antiapoptotic effect was obtained by Ca^2+^_cyt_ chelating using BAPTA. These data indicate that NUP-induced cell death is mediated, at least in part, by the induction of oxidative stress and Ca^2+^_cyt_ accumulation. However, a substantial apoptotic activity of pure DTBN (0.05–0.25 µg/mL), was found to be independent of cytosolic ROS or Ca^2+^, suggesting that alternative mechanisms are involved in DTBN-induced cytotoxicity. Notably, neither NUP nor DTBN treatment significantly induced cell death of normal human peripheral blood mononuclear cells. Our results provide the basis for further investigation of the antileukemic potential of NUP and its active constituents.

## 1. Introduction

Acute myeloid leukemia (AML) is a highly aggressive blood cancer characterized by enhanced clonal proliferation and impaired differentiation of myeloid progenitors. The currently available therapy is noncurative for most patients with AML, an average 5-year overall survival rate being about 24% [1]. Thus, it is of great significance to augment the existing drug armamentarium with new agents that can increase the life span of patients with AML. Indeed, several novel targeted AML drugs and formulations of existent drugs have recently been approved by the U.S. Food and Drug Administration (FDA) [2] and are currently used in clinic. Still, long-term survival benefits of these drugs or their combinations have yet to be determined [3].

Numerous studies have demonstrated anticancer effects of various phytochemicals in preclinical models of neoplastic diseases, including hematological malignancies (reviewed in [4,5,6,7]). The rationale for potential therapeutic applications of these agents is based on their pleiotropic cellular effects, including the ability to induce oxidative stress (e.g., [8]) in various cancer cell types which results in cell growth arrest and cell death. We (e.g., [9,10,11]) and others (see [6,12,13] for recent reviews) have demonstrated anticancer effects of different plant extracts and purified phenolic compounds as well as their ability to cooperate with one another. Particularly, we have recently reported that the polyphenol curcumin or the phenolic acid ester methyl 4-hydroxycinnamate can synergize with the phenolic diterpene carnosic acid to induce massive apoptosis in AML cells via sustained accumulation of the cytosolic Ca^2+^ (Ca^2+^_cyt_), without inducing oxidative stress [14,15].

A number of naturally occurring alkaloids and their derivatives are currently in use as cancer therapeutics. Among these are the *Vinca* alkaloids vincristine and vinblastine approved for lymphocytic leukemias and lymphomas [16], homoharringtonine for chronic myeloid leukemia [17], and the cyclopamine derivative glasdegib for AML [18]. We have previously reported a marked in vitro cytotoxicity and proapoptotic activity of the sesquiterpene thioalkaloid-enriched fraction (termed NUP) of the methanolic extract from the leaves of yellow water lily *Nuphar lutea* (L.) Sm. [19]. NUP was also shown to potentiate the antimetastatic effect of cisplatin in the murine B16 melanoma lung metastasis model in vivo [20]. Dimeric sesquiterpene thioalkaloids with the 6-hydroxyl group purified from *Nuphar* species, e.g., 6-hydroxythiobinupharidine, 6,6′-dihydroxythiobinupharidine (DTBN), and 6-hydroxythionuphlutine B (Supplementary Figure S1), have been shown to exhibit strong apoptosis-inducing effects on several types of cancer cells, including AML cell lines [19,21,22,23]; however, the molecular mechanisms underlying the cytotoxicity of these compounds remain largely unknown.

In the present study, we examined the in vitro antileukemic activity of NUP in human AML cell lines. Our data demonstrate that NUP treatment resulted in a reduction in proliferation and viability of AML cells that was associated with caspase-dependent apoptosis induction. NUP-induced cell death was mediated, at least in part, by the induction of oxidative stress and Ca^2+^_cyt_ accumulation. However, while pure DTBN demonstrated even a stronger apoptosis-inducing activity, this effect was found to be independent of the cytosolic reactive oxygen species (ROS) or Ca^2+^. Importantly, neither NUP nor DTBN significantly induced cell death or reduced the cell viability of normal human peripheral blood mononuclear cells at the concentrations that killed AML cells.

## 2. Results

### 2.1. Thioalkaloid-Enriched Fraction from the N. lutea Leaf Extract (NUP) Inhibits the Growth of AML Cells

**Figure 1:**
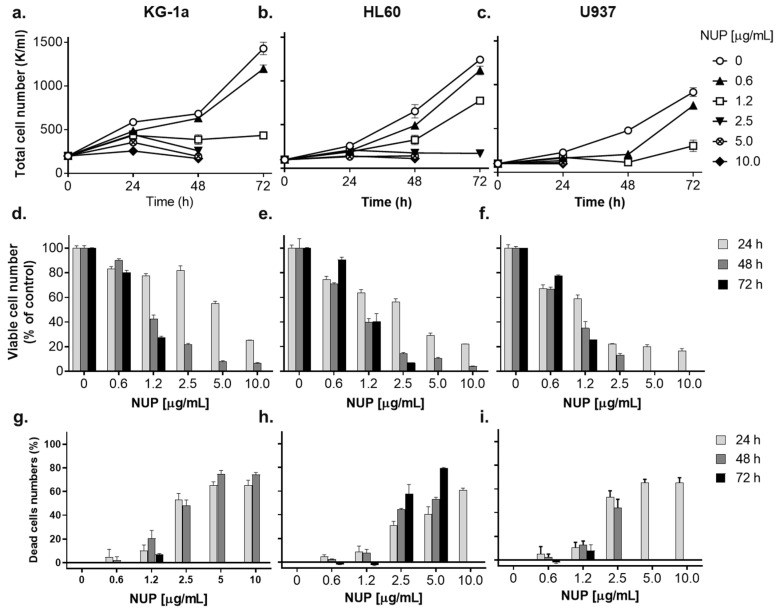
Thioalkaloid-enriched fraction from the *N. lutea* leaf extract (NUP) induces growth inhibition and cytotoxicity in AML cell cultures. KG-1a HL60 and U937 cells (1.5 × 10^5^ cells/mL) were incubated with vehicle or NUP at the indicated concentrations for 24–72 h, followed by the Trypan Blue exclusion assay. (**a**–**c**) Changes in the total (viable *plus* dead) cell numbers; (**d**–**f**) changes in the viable cell numbers; (**g**–**i**) changes in the percentage of dead cells. The data are means ± SD of at least 3 independent experiments performed in triplicate.

To determine the effects of NUP on the growth of AML cell cultures, exponentially growing KG-1a, HL60, and U937 human AML cells were incubated with increasing concentrations of NUP (0.3–10 µg/mL) for 24, 48, and 72 h. Viable and dead cells were then enumerated using the Trypan Blue exclusion assay. The data demonstrate that NUP strongly inhibited the growth of the three cell lines tested, as indicated by the concentration- and time-dependent reduction in the total (viable + dead) cell numbers (Figure 1a–c). This was associated with a corresponding decrease in the numbers of viable cells (Figure 1d–f and Table 1). Interestingly, the marked reduction in the total and viable cell numbers caused by lower NUP concentrations (up to 1.2 µg/mL) was accompanied by only a slight increase in cell death (Figure 1g–i), indicative of an antiproliferative rather than a cytotoxic effect. Higher NUP concentrations (2.5–10.0 µg/mL) produced a substantial concentration- and time-dependent increase in the percentage of dead cells (Figure 1g–i). The KG-1a and, particularly, the U937 cell line demonstrated the highest susceptibility to NUP-induced cytotoxicity. For instance, the total loss of countable U937 cells was observed as early as at 48 h of incubation with 5–10 µg/mL NUP (Figure 1c,f,i). The order of the sensitivity of the cell lines to NUP, particularly at 24 h, was U937 > HL60 > KG-1a (see Figure 1 and Table 1 for IC_50_ values). Generally, all three cell lines exhibited a strong reduction in both the cell number and cell viability at 5 µg/mL and 10 µg/mL NUP.

### 2.2. NUP Induces Caspase-Dependent Apoptosis

**Figure 2:**
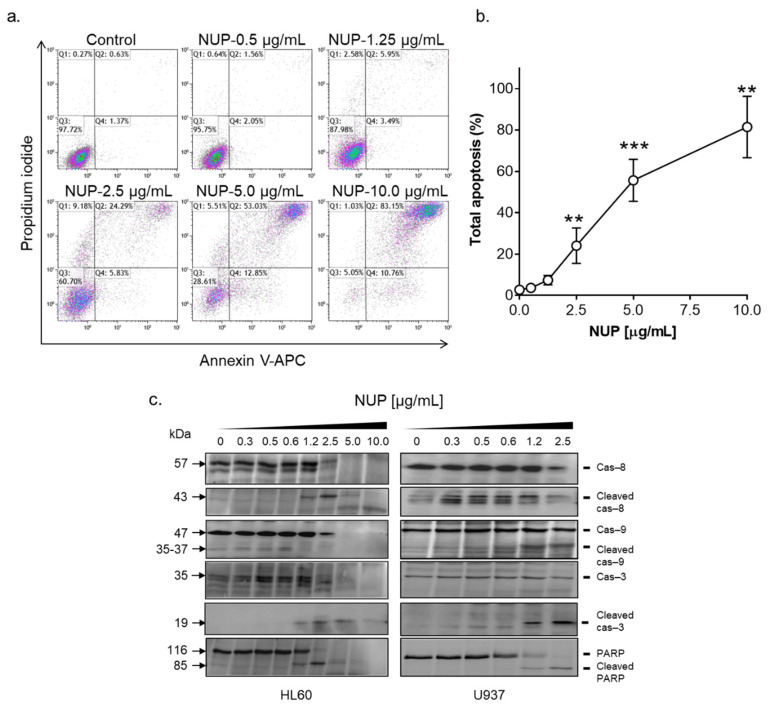
NUP induces apoptosis in AML cells. HL60 and U937 cells were treated with vehicle or NUP at the indicated concentrations, for 24 h. (**a**,**b**) The extent of apoptosis was measured by the annexin-V/PI assay in HL60 cells; (**a**) Typical flow cytometric data obtained in a representative experiment. (**b**) Averaged percentages of apoptotic (early + late) cells. The data are means ± SD of at least 3 independent experiments performed in duplicate. **, *p* < 0.01 and ***, *p* < 0.001 vs. vehicle-treated (control) cells; (**c**) Western blot analysis of caspase (Cas) and PARP cleavage in HL60 and U937 cells treated with increasing concentrations of NUP, for 24 h. Representative blots of 3 independent experiments are shown.

**Figure 3:**
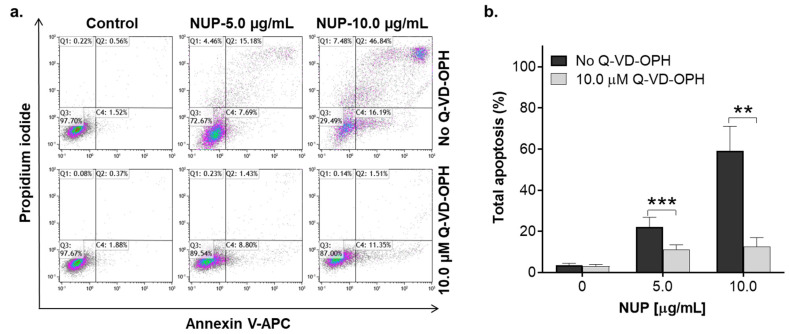
NUP-induced apoptotic effect is caspase-dependent. HL60 cells were pre-incubated with vehicle or the pan-caspase inhibitor Q-VD-OPH (10 µM) for 1 h followed by treatment with vehicle or NUP (5 µg/mL and 10 µg/mL) for another 24 h. The extent of apoptosis was measured by the annexin-V/PI assay. (**a**) Typical flow cytometric data obtained in a representative experiment. (**b**) Averaged percentages of apoptotic (early + late) cells. The data are means ± SD of 3 independent experiments performed in duplicate. **, *p* < 0.01 and ***, *p* < 0.001, significant differences between the indicated groups.

To elucidate the mode of the pronounced cytotoxicity of NUP, we first performed the annexin-V/PI binding assay in HL60 cells. As shown in Figure 2a,b, exposure of HL60 cells to 0.5–10 µg/mL NUP for 24 h resulted in a concentration-dependent increase in the extent of apoptosis induction. Consistent with the cytotoxicity data (Figure 1g–i), significant apoptotic cell death was observed at 2.5–10 µg/mL NUP. Particularly, most of the cells were found to be annexin-V/PI double-positive, indicative of the late apoptosis phase, while minor populations (5–15%) of early apoptotic (annexin-V-positive/PI-negative) or supposedly necrotic (annexin-V-negative/PI-positive) cells were also detected (Figure 2b). Detailed analysis of NUP-induced apoptosis in AML cells revealed that the appearance of annexin-V/PI-positive cells was accompanied by caspase-3 and PARP cleavage (Figure 2c). It was also found that NUP treatment of HL60 and U937 cells led to the activation of both initiator caspase-8 and caspase-9 (Figure 2c), which are involved in the extrinsic and intrinsic apoptotic pathway, respectively. To determine whether NUP induced apoptosis is caspase-dependent, we used the pan-caspase inhibitor Q-VD-OPH and found that, while having no effect when added alone, it dramatically reduced the apoptotic effect of NUP at 5 µg/mL and 10 µg/mL (Figure 3). Taken together, the above results indicate that NUP treatment kills AML cells primarily by a caspase-mediated apoptosis induction.

### 2.3. Involvement of Reactive Oxygen Species in NUP-Induced Apoptosis

**Figure 4:**
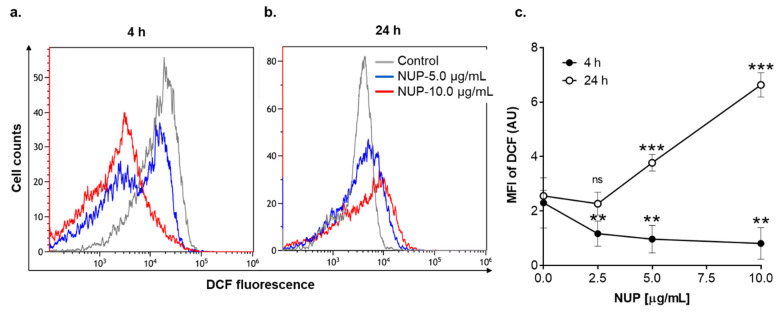
NUP induces time-dependent biphasic changes in the cytosolic levels of reactive oxygen species as well as a decrease in GSH levels after a longer treatment. Following incubation with vehicle or indicated concentrations of NUP, HL60 cells were loaded with DCFH-DA followed by flow cytometric analysis, as described in Materials and Methods. (**a**,**b**) Typical examples of cytosolic ROS measurement at the indicated time points. (**c**) Averaged relative ROS levels expressed by DCF geometric mean fluorescence intensity (MFI) units, as determined after 4 h and 24 h of incubation; the data are means ± SD of 3 independent experiments performed in duplicate. **, *p* < 0.01 and ***, *p* < 0.001 vs. vehicle-treated (control) cells; ns, non-significant.

**Figure 5:**
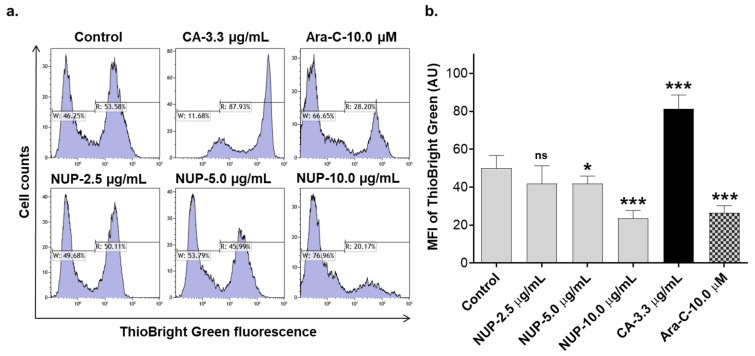
NUP induces a decrease in cellular GSH levels after a longer treatment. Following treatment with vehicle or indicated concentrations of NUP, for 24 h, HL60 cells were loaded with ThioBright Green followed by flow cytometric analysis, as described in Materials and Methods. (**a**) Typical examples of cellular GSH measurement from a representative experiment. (**b**) Averaged GSH levels expressed as ThioBright Green geometric mean fluorescence intensity (MFI) units. The data are means ± SD of 3 independent experiments performed in duplicate. *, *p* < 0.1 and ***, *p*< 0.001 vs. vehicle-treated (control) cells; ns, non-significant. CA, carnosic acid; AraC, 1-β-d-arabinosylcytosine.

**Figure 6:**
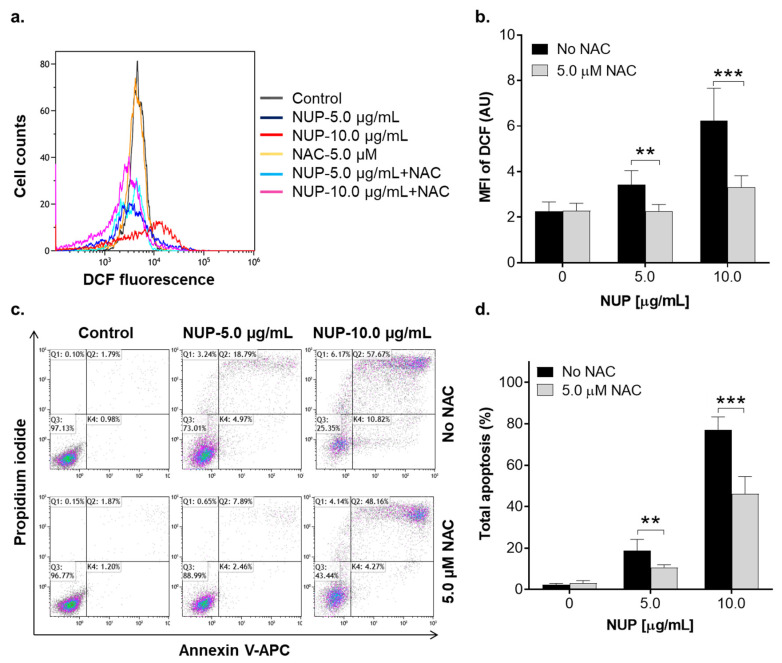
The thiol antioxidant N-acetylcysteine abrogates ROS accumulation and partially inhibits apoptosis induction in NUP-treated cells. HL60 cells were pre-incubated with 5 µM NAC for 1 h followed by the addition of 5 µg/mL or 10 µg/mL NUP for another 24 h. Cytosolic ROS levels and the extent of apoptosis were then measured, as described above. (**a**) Typical examples of cytosolic ROS measurement from a representative experiment. (**b**) Averaged ROS levels expressed as DCF geometric mean fluorescence intensity (MFI) units. (**c**) Typical annexin-V/PI binding data obtained in a representative experiment. (**d**) Averaged percentages of apoptotic (early + late) cells. The data are means ± SD of 3 independent experiments performed in duplicate. **, *p* < 0.01 and ***, *p* < 0.001, significant differences between the indicated groups.

We have previously reported that DTBN, one of the sesquiterpene thioalkaloids purified from *N. lutea*, augments ROS production by differentiated neutrophil-like HL60 cells [24]. Here, we examined the effect of NUP on cytosolic ROS levels in undifferentiated HL60 cells by flow cytometry using the oxidation-sensitive fluorescent probe DCFH-DA. Treatment with cytotoxic NUP concentrations (2.5–10.0 µg/mL) resulted in significant biphasic changes in ROS levels relative to those in untreated cells—a decrease at an early time point (4 h; Figure 4a,c) followed by an increase after a longer incubation for 24 h (Figure 4b,c). ROS accumulation observed at 24 h was accompanied by a significant concentration-dependent decrease in the cellular GSH level (Figure 5). The effect of the highest concentration of NUP (10 µg/mL) was similar to that of the AML chemotherapeutic drug cytosine arabinoside (AraC; 10 µM), while exposure to the plant polyphenolic antioxidant carnosic acid at 3.3 µg/mL (10 µM), used as the positive control, resulted in a marked increase in the cellular GSH level (Figure 5). Similar to NUP, treatment with AraC for 24 h markedly reduced cell viability (by 70–80%), whereas carnosic acid had no cytotoxic effect whatsoever (data not shown; see also Ref. [11]). To determine whether NUP-induced cell death is mediated by enhanced ROS generation, we incubated HL60 cells with 5 µg/mL or 10 µg/mL NUP for 24 h in the absence or presence of the thiol antioxidant and glutathione precursor N-acetyl cysteine (NAC). As shown in Figure 6a,b, co-incubation with NAC almost completely abrogated cytosolic ROS elevation at both 5 µg/mL and 10 µg/mL NUP and markedly, though partially (by 30–50%), inhibited apoptosis induction in HL60 cells (Figure 6c,d). Collectively, these data indicate that in AML cell cultures, NUP-induced cell death is, at least in part, mediated by the induction of oxidative stress.

### 2.4. The Role of Changes in Ca^2+^_cyt_ Levels in NUP-Induced Apoptosis

**Figure 7:**
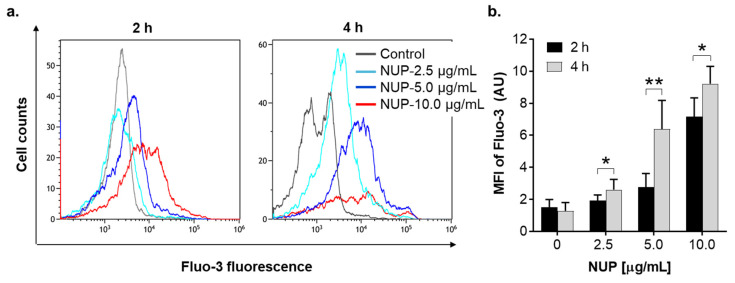
Sustained elevation of Ca^2+^_cyt_ in NUP-treated HL60 cells. Cells were incubated with the indicated NUP concentrations for 2 h or 4 h. (**a**) Typical examples of Ca^2+^_cyt_ measurement from a representative experiment. (**b**) Averaged relative Ca^2+^_cyt_ levels expressed as Fluo-3 geometric mean fluorescence intensity (MFI) units. The data are means ± SD of 3 independent experiments performed in duplicate. *, *p* < 0.1 and **, *p* < 0.01, significant differences between the indicated groups.

**Figure 8:**
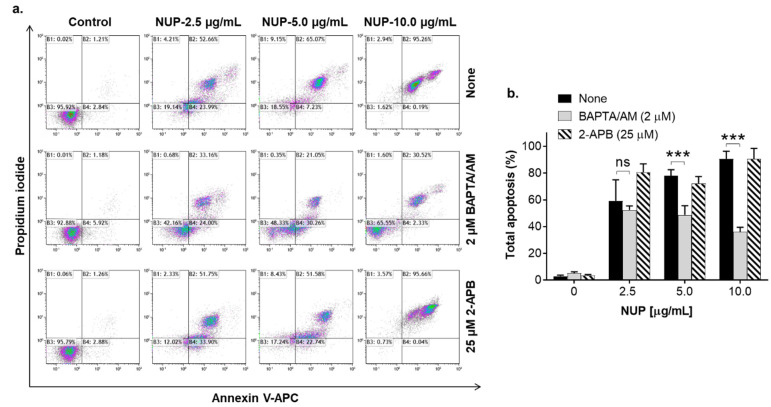
NUP-induced apoptosis is partially mediated by Ca^2+^_cyt_ rise. HL60 cells were preincubated with BAPTA/AM (1 µM) or 2-APB (25 µM) for 1 h, followed by treatment with the indicated NUP concentrations for 24 h. The extent of apoptosis was measured by the annexin-V/PI assay. (**a**) Typical flow cytometric data from a representative experiment; (**b**) Averaged percentages of apoptotic (early + late) cells. The data are means ± SD of 3 independent experiments performed in duplicate. ***, *p* < 0.001, significant differences between the indicated groups; ns, non-significant.

To determine whether NUP-induced apoptosis is associated with elevation of Ca^2+^_cyt_, we first measured steady-state Ca^2+^_cyt_ levels in HL60 cells using the fluorescent Ca^2+^ indicator Fluo-3. Cells were incubated with NUP at 2.5 µg/mL, 5 µg/mL, or 10 µg/mL for 2 h and 4 h followed by flow cytometric analysis. The results demonstrated that NUP treatment caused sustained Ca^2+^_cyt_ rise up to ~10-fold of the basal level in a time- and concentration-dependent manner (Figure 7), suggesting that this effect may contribute to NUP-induced cytotoxicity. To test this hypothesis, HL60 cells were preloaded with the intracellular Ca^2+^ chelating agent BAPTA/AM (1 µM) or the inositol trisphosphate receptor (IP_3_R) and store-operated calcium entry (SOCE) blocker 2-aminoethoxydiphenyl borate (2-APB; 25 µM) followed by incubation with 2.5–10 µg/mL NUP for 24 h. Intracellular Ca^2+^ chelation significantly attenuated apoptosis induction depending on NUP concentration—the higher the NUP concentration applied, the stronger the antiapoptotic effect was observed in BAPTA-loaded cells (Figure 8a,b). On the other hand, 2-APB had no effect whatsoever on NUP-induced apoptosis (Figure 8a,b). These data suggest that the apoptotic effect of NUP was at least in part mediated by Ca^2+^_cyt_ rise, but likely not via the IP_3_R/SOC activation.

### 2.5. Comparison of the Proapoptotic Effects of NUP and Purified 6,6′-Dihydroxythiobinupharidine

**Figure 9:**
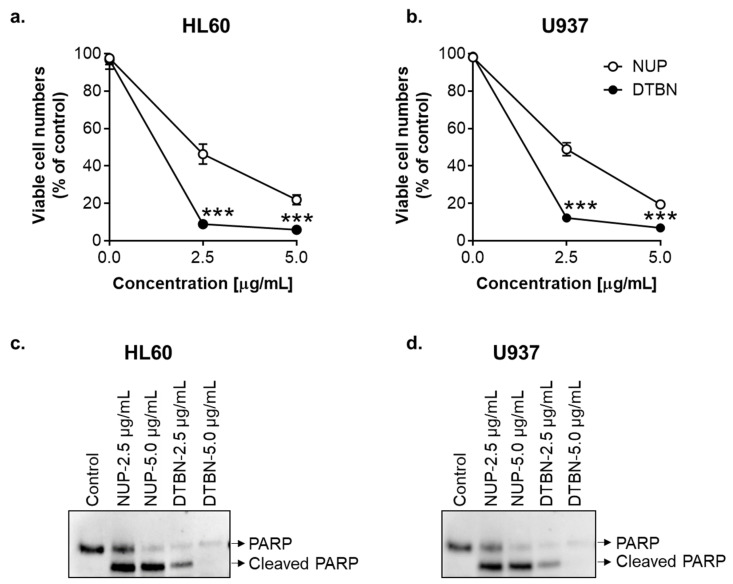
Comparison of the effects of NUP and purified 6,6′-dihydroxythiobinupharidine (DTBN) on the number of viable cells and apoptosis induction. HL60 (**a**,**c**) and U937 (**c**,**d**) cells were treated with NUP or DTBN (2.5 µg/mL and 5 µg/mL) followed by cell enumeration or protein expression analysis, as indicated below. (**a**,**b**) Effects of the indicated agents on the number of viable cells following 24 h of incubation, as determined by the Trypan Blue exclusion assay. The data are means ± SD of at least 3 independent experiments performed in triplicate; ***, *p* < 0.001, vs. vehicle-treated (control) cells; (**c**,**d**) Western blot analysis of PARP cleavage following 4 h of incubation. Representative blots of 3 independent experiments are shown. DTBN, 6,6′-dihydroxythiobinupharidine.

It has been reported that dimeric sesquiterpene thioalkaloids with the 6-hydroxyl group, such as 6-hydroxythiobinupharidine, 6,6′-dihydroxythiobinupharidine (DTBN), and 6-hydroxythionuphlutine B, produce strong cytotoxic effects on U937 and NB4 AML cells at concentrations of 1–10 µM [21,23]. Furthermore, DTBN was shown to rapidly (within 1–6 h) induce apoptosis in these cells [21,23]. As NUP contains all these and several other dimeric sesquiterpene thioalkaloids, we directly compared the in vitro antileukemic effects of NUP and pure DTBN using HL60 and U937 cells as models. For the cell viability test, cells were treated with 2.5 µM and 5.0 µM NUP or DTBN, for 24 h followed by the Trypan Blue exclusion assay. As shown in Figure 9a,b, the two agents concentration-dependently reduced the number of viable cells in both cell lines; however, DTBN was significantly more effective compared to NUP, particularly at the lower concentration (2.5 µg/mL). To compare the apoptosis-inducing effects of NUP and DTBN, we determined PARP cleavage in HL60 cells and U937 cells following 4 h of incubation using Western blot analysis. The results indicated that DTBN was again more potent compared to NUP as seen by a complete PARP cleavage at 2.5 µg/mL DTBN and even disappearance of the cleaved 86-kD fragment at the higher concentration (5 µg/mL) in both HL60 and U937 cells (Figure 9c,d).

**Figure 10:**
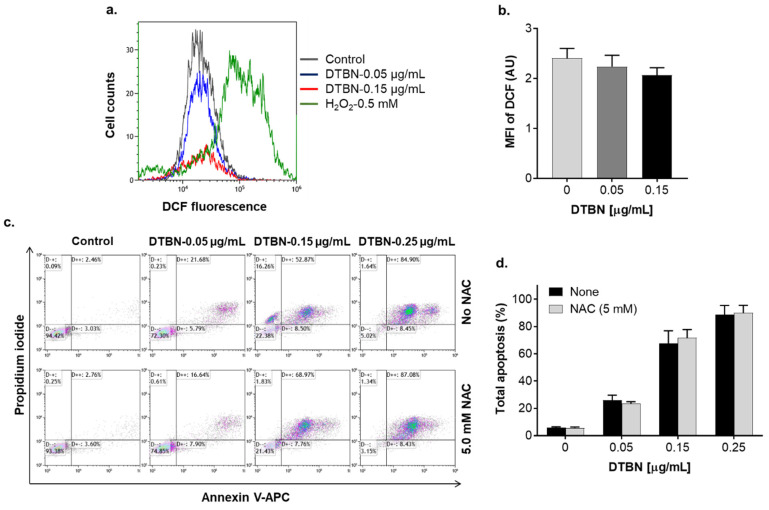
6,6′-dihydroxythiobinupharidine (DTBN)-induced apoptosis is not mediated by the cytosolic ROS. HL60 cells were pre-incubated with or without 5 µM NAC for 1 h followed by the addition of the indicated concentrations of DTBN for another 24 h. The extent of apoptosis and cytosolic ROS levels were measured using the annexin-V/PI assay and the cytosolic ROS indicator DCFH-DA, respectively. (**a**) Typical flow cytometric data from a representative annexin-V/PI assay. (**b**) Averaged percentages of apoptotic (early + late) cells. (**c**) Typical examples of cytosolic ROS measurement from a representative experiment. H_2_O_2_ (0.5 mM) was used as the positive control. (**d**) Averaged ROS levels expressed as DCF geometric mean fluorescence intensity (MFI) units. The data are means ± SD of 3 independent experiments performed in duplicate.

**Figure 11:**
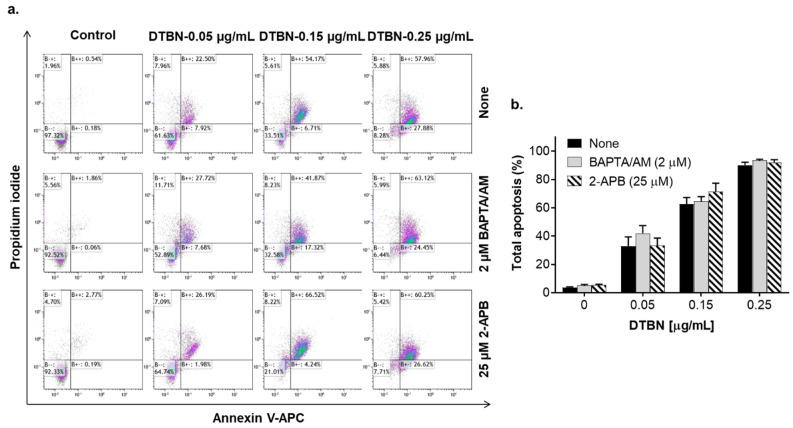
6,6′-dihydroxythiobinupharidine (DTBN)-induced apoptosis is not mediated by Ca^2+^_cyt_. HL60 cells were preincubated with BAPTA/AM (1 µM) or 2-APB (25 µM) for 1 h, followed by treatment with the indicated concentrations of DTBN for 24 h. The extent of apoptosis was measured by the annexin-V/PI assay. (**a**) Typical flow cytometric data from a representative experiment; (**b**) Averaged percentages of apoptotic (early + late) cells. The data are means ± SD of 3 independent experiments performed in duplicate.

We then explored possible roles of the cytosolic ROS and Ca^2+^ in DTBN-induced apoptosis using HL60 cells as a model. Incubation with low concentrations of DTBN (0.05–0.25 µg/mL) for 24 h resulted in a concentration-dependent induction of apoptosis in 30–90% DTBN-treated cells, as determined by the annexin-V/PI assay (Figure 10c,d and Figure 11). Unexpectedly, in contrast to NUP (Figure 4), DTBN-induced apoptosis observed 24 h posttreatment was not accompanied by elevation of the cytosolic ROS levels, as demonstrated by measuring cellular DCF fluorescence (Figure 10a,b). On the other hand, H_2_O_2_, used as the positive control, induced a pronounced increase in ROS levels in the same experiments (Figure 10a). Furthermore, unlike in NUP-treated cells (Figure 6c,d), the extent of DTBN-induced apoptosis was not significantly altered in the presence of the antioxidant NAC (Figure 10c,d). Likewise, in contrast to NUP (Figure 8), the apoptotic effect of DTBN was not significantly affected by the intracellular Ca^2+^ chelator BAPTA, and 2-APB was as inefficient as in NUP-treated cells (Figure 11a,b). These results suggest that DTBN-induced apoptosis in HL60 cells is not mediated by the cytosolic ROS or Ca^2+^.

### 2.6. Effects of NUP and Purified 6,6′-Dihydroxythiobinupharidine on Cell Death and Viability of Peripheral Blood Mononuclear Cells from Healthy Donors

**Figure 12:**
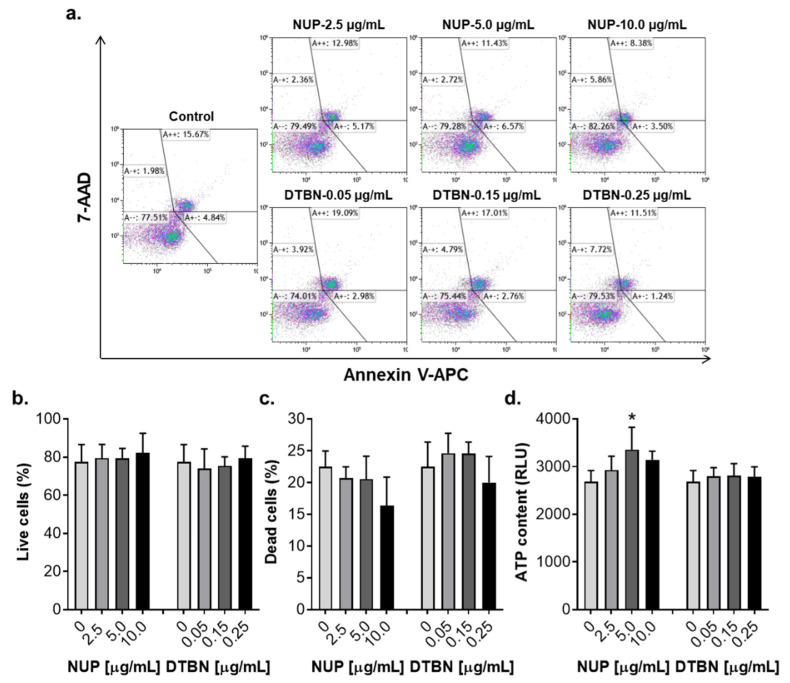
Effects of NUP and 6,6′-dihydroxythiobinupharidine (DTBN) on cell death and viability of normal human peripheral blood mononuclear cells (PBMC). Cells (1.5 × 10^5^/mL) were incubated with the indicated concentrations of NUP or DTBN for 24 h. The extent of cell death and viability was measured by the annexin-V/PI assay and the ATP assay, respectively. (**a**) Typical flow cytometric data from a representative experiment. (**b**) Averaged percentages of live (annexin-V/PI double-negative) cells. (**c**) Averaged percentages of dead (annexin-V only-positive *plus* PI only-positive *plus* annexin-V/PI double-positive) cells. (**d**) Changes in cell viability measured on the basis of a relative cellular ATP content. The data are means ± SD of 3 independent experiments performed in duplicate (**b**,**c**) or quadruplicate (**d**). *, *p* < 0.001, vs. vehicle-treated (control) cells.

Most phytochemicals are multitargeted and pleiotropically acting compounds which may affect various cell types. Therefore, it was important to determine whether, similar to AML cells, NUP and DTBN would kill normal human white blood cells in the same concentration ranges. We thus measured the effects of these agents on cell death (by the annexin-V/PI assay) and viability (by the ATP assay) of human peripheral blood mononuclear cells (PBMC) isolated from healthy donors. Notably, in contrast to AML cell cultures which displayed dramatic reductions in the live (annexin-V/PI-double negative) cell population in response to 2.5–10.0 µg/mL NUP or 0.05–0.25 µg/mL DTBN (e.g., Figure 2a and Figure 10c), no significant decrease in the percentage of live cells was observed in PBMC cultures treated with either agent for 24 h, as compared to control (Figure 12a,b). As shown in Figure 12a,c, incubation of PBMC samples with vehicle resulted in the appearance of 20–25% dead cells. However, there was no significant elevation in the relative total percentage of dead (annexin-V only-positive *plus* PI only-positive *plus* annexin-V/PI-double positive) cells, which even tended to decrease following treatment with 10.0 µg/mL NUP (Figure 12c). This correlated with a significant increase in PBMC viability in NUP-treated cultures, as measured on the basis of the cellular ATP content (Figure 12d). Treatment with DTBN did not affect PBMC viability (Figure 12d).

## 3. Discussion

*N. lutea* and other *Nuphar* species have been widely used in traditional medicine [25,26,27,28]. Plant extracts of these species are rich in alkaloids and polyphenolic compounds which are of potential therapeutic value. Indeed, the therapy-related actions discovered in recent studies of *N. lutea* extracts and individual sesquiterpene thioalkaloids (summarized in [29]) include anti-inflammatory, antibacterial [30,31], antiviral [32], antifungal [33], antiparasitic [34], and anticancer [19,20,21,22,23] activities.

In this study, we characterized the in vitro antileukemic effects of the thioalkaloid-enriched fraction of *N. lutea* leaf extract (NUP) [19] in comparison with those produced by purified 6,6′-dihydroxythiobinupharidine (DTBN). NUP was found to cause a concentration- and time-dependent inhibition of cell growth, cytotoxicity, and caspase-dependent apoptosis induction in AML cell cultures. Interestingly, AML cell lines representing different stages of myeloid maturation—KG-1a (leukemia stem-like cells), HL60 (myeloblastic leukemia), and U937 (myelomonocytic leukemia)—displayed differential sensitivity toward NUP-induced cytotoxicity, the least differentiated KG-1a cells being relatively the most resistant among the cell lines tested (Figure 1 and Table 1). This dependence on the cell maturation status is intriguing and requires further investigation.

Pure DTBN exhibited a much higher proapoptotic potency relative to NUP (Figure 9; also compare Figure 2a,b and Figure 10c,d). This is consistent with the previously reported data showing that DTBN and other dimeric sesquiterpene thioalkaloids with the 6-hydroxyl group, such as 6-hydroxythiobinupharidine and 6-hydroxythionuphlutine B, induce substantial and rapid apoptotic cell death of AML cells at low concentrations [21,23]. It is possible that all these thioalkaloids as well as other phytochemical constituents of the *N. lutea* methanolic extract contribute, to a varying extent, to the cytotoxic and apoptosis-inducing effects of NUP. Importantly, in contrast to AML cells, treatment of normal human PBMC with either NUP or DTBN did not induce cell death or reduce cell viability (Figure 12). Collectively, these data suggest that DTBN and other NUP constituents may have selective antileukemic activities. However, more studies are required to test this suggestion in a wide range of leukemia and normal cell types.

As studies of the molecular mechanisms underlying the cytotoxicity of *Nuphar* thioalkaloids have only recently been initiated (e.g., [35]), our primary objective was to determine the possible roles of the intracellular ROS and Ca^2+^ in the induction of apoptosis by NUP and DTBN. Various phytochemicals have been shown to induce cancer cell death through the generation of oxidative stress mediated by the accumulation of ROS and/or suppression of cellular antioxidant defense capacity, e.g., glutathione depletion (e.g., [36,37]). In our mechanistic experiments, we used HL60 myeloblastic leukemia cells which, as stated above, displayed medium sensitivity to NUP compared to more primitive KG-1a cells and more mature U937 cells (Figure 1 and Table 1). Cell treatment with NUP resulted in a time-dependent biphasic effect on the cytosolic ROS levels—an antioxidant effect at an early time point (4 h) and a prooxidant effect at a longer incubation (24 h). Interestingly, both types of changes in the ROS status were associated with the induction of apoptosis, as demonstrated by both a substantial PARP cleavage at 4 h (Figure 9c,d) and a markedly increased annexin-V/PI binding at 24 h (Figure 2 and Figure 3), suggesting that in NUP-treated cells, ROS do not play a role in the initiation of apoptosis but may contribute to the apoptosis-related cell damage at later stages. The latter effect, which is partially prevented by the thiol antioxidant NAC (Figure 6), may be due, in part, to the glutathione-depleting action of NUP (Figure 5).

The proapoptotic effects of different phytochemicals, such as the sesquiterpene lactone thapsigargin that is a specific inhibitor of sarco/endoplasmic reticulum Ca^2+^-ATPase [38], and plant polyphenols [39,40,41,42] on cancer cells have been associated with the alteration of the intracellular Ca^2+^ homeostasis. We have recently reported that the combinations of curcumin or methyl 4-hydroxycinnamate with carnosic acid are capable of inducing apoptotic cell death of AML cells solely by triggering sustained Ca^2+^_cyt_ accumulation, without inducing oxidative stress [14,15,43]. Here, we show that NUP-induced apoptosis is partially mediated by an increase in Ca^2+^_cyt_ levels because this apoptotic effect was attenuated in cells preloaded with the intracellular Ca^2+^ chelator BAPTA (Figure 11). Interestingly, in contrast to the above combinations of plant phenolic agents [14,15], the IP_3_R/SOCE blocker 2-APB did not significantly modulate NUP-induced apoptosis, suggesting that in this case the intracellular Ca^2+^ mobilization or the extracellular Ca^2+^ entry into NUP-treated cells occurred via other routes. For instance, there is evidence that Ca^2+^ influx evoked by the polyphenol gossipol in endothelial cells is mediated by the transient receptor potential cation vallinoid (TRPV) channels [44]. Another polyphenol, resveratrol, was found to activate another type of the TRP (ankyrin 1) channels (TRPA1) in cancer-associated fibroblasts [45].

The lack of the prooxidant action of purified DTBN (Figure 10a,b) and the inability of NAC (Figure 10c,d) or BAPTA (Figure 11) to attenuate DTNB-induced apoptosis raises the possibility that the partial ROS and Ca^2+^ dependence of the proapoptotic activity of NUP is attributed to its other component(s), such as additional dimeric sesquiterpene thioalkaloids or polyphenolic compounds, rather than DTBN. The possible mechanisms of DTBN-induced apoptosis may involve its recently discovered ability to inhibit type II topoisomerase [35], one of the major molecular targets for cytotoxic cancer therapy (e.g., [46]). Another recent study by our group has shown that DTBN can act as an inhibitor of classical protein kinase C (PKC) isoenzymes [47], which may also significantly contribute to its proapoptotic effect (e.g., [48]). In addition, we have reported that DTBN is capable of inhibiting cathepsins (cysteine proteases) in a clear type-specific manner, cathepsin S being the most susceptible compared to cathepsins B, L or another cysteine protease papain [29]. Docking simulations revealed that the cysteine sulfur of the proteases was in close proximity to the DTNB thiaspirane ring, potentially forming the necessary conditions for a nucleophilic attack to form a disulfide bond [29]. Downregulation of cathepsin S was previously reported to promote apoptotic cell death in hepatocellular carcinoma [49] and glioblastoma [50] cells. Therefore, the proapoptotic activity of nupharidines may be related to their electrophilicity and selective protein thiol targeting. Further studies are required to pinpoint the observed antileukemic actions of NUP to specific chemical components of the *N. lutea* extract, and to elucidate the precise mechanisms underlying the apoptosis-inducing activity of DTBN and other *Nuphar* thioalkaloids.

### 3.1. Materials

Intracellular glutathione (GSH) Assay Kit was from ImmunoChemistry Technologies (Bloomington, MN, USA), and Quinoline-Val-Asp-Difluorophenoxymethylketone (Q-VD-OPH) was from Cayman Chemical (Ann Arbor, MI, USA). 6,6′-dihydroxythiobinupharidine (DTBN), propidium iodide (PI), *N*-acetyl-l-cysteine, 1-β-d-arabinosylcytosine (AraC), and 2-aminoethoxydiphenyl borate (2-APB) were from Merck-Sigma-Aldrich (Rehovot, Israel). Annexin-V-APC was obtained from BioLegend (San Diego, CA, USA). 2′,7′-dichlorofluorescein-diacetate (DCFH-DA) and bis-(*o*-aminophenoxy)-ethane-*N*,*N*,*N*’,*N*’-tetraacetic acid, tetra(acetoxymethyl)-ester (BAPTA) were purchased from Santa Cruz Biotechnology (Dallas, TX, USA). Hank’s buffered salt solution (HBSS), Ca^2+^/Mg^2+^-free phosphate buffered saline (PBS), penicillin, streptomycin, and HEPES buffer were purchased from Biological Industries (Beth Haemek, Israel). RPMI 1640 medium and heat-inactivated fetal bovine serum (FBS) were purchased from Gibco-Invitrogen (Carlsbad, CA, USA).

### 3.2. Preparation of Nuphar lutea Extract and Its Fractions Containing Sesquiterpene Thioalkaloids

*N. lutea* extract was prepared and fractionated as detailed previously [19,32]. Briefly, floating and submerged leaves of *N. lutea* were collected, oven dried, and extracted with methanol. The extract was dried by evaporation and the residue was fractionated on a silica gel column using chloroform/ethyl-acetate/diethylamine (20:1:1, *v*/*v*/*v*) as a solvent. Fractions were monitored using a previously generated L428 Hodgkin’s lymphoma cell line stably expressing the NF-κB-luciferase reporter construct [19,32]. Fractions producing the strongest inhibitory effects on NF-κB reporter activity were pooled, dried, re-dissolved in DMSO at the concentration of 10 mg/mL, and stored at −20 °C. This pool is termed “NUP” and its preparation was standardized previously [19,32]. NUP samples were analyzed by one-dimensional and two-dimensional NMR spectroscopy. The ^1^H and ^13^C NMR spectra indicated the presence of several dimeric sesquiterpene thioalkaloids as the major NUP constituents (see Ref. [19] and Supplemental Figure S1 and Table S2 therein as well as Ref. [32] and Figure 6 therein).

### 3.3. Cell Culture and Enumeration

AML cell lines—HL60 (CCL-240), U937 (CRL-1593.2), and KG-1a (CCL-246.1)—were obtained from the American Type Culture Collection (Rockville, MD, USA). Cells were cultured in RPMI 1640 medium supplemented with 10% FBS, penicillin (100 U/mL), streptomycin (0.1 mg/mL), and 10 mM HEPES (pH = 7.4) at 37 °C in a 5% CO_2_ environment. For cell counting, HL60, U937, and KG-1a cells (4–8 × 10^4^/mL) were plated in 24-well plates and incubated with increasing concentrations of NUP for 24–72 h. The number of live and dead cells was determined on the basis of the trypan blue exclusion assay by counting in Vi-Cell XR cell viability analyzer (Beckman Coulter, Fullerton, CA, USA).

Samples of peripheral blood were collected with informed consent from healthy adult donors upon the approval No. 0328-16-SOR by the institutional Helsinki committee (Soroka University Medical Center, Beer Sheva, Israel). Peripheral blood mononuclear blood cells (PBMCs) were isolated as described previously [14,15] using Histopaque-1077 gradient centrifugation and stored in liquid N_2_. Immediately before experiment, cell samples were unfrozen, washed in RPMI supplemented with 20% BSA, 10 mM HEPES (pH = 7.4), 2 mM L-glutamine, 100 U/mL penicillin, 0.1 mg/mL streptomycin, 0.01 µg/mL insulin, 5 µg/mL *holo*-transferrin, and were then incubated at 1.5 × 10^5^ cells/mL with test agents in the same medium, for 24 h at 37 °C in a 5% CO_2_ environment. Cells were collected and analyzed by the annexin-V/propidium iodide binding assay (see Section 3.4 below) and cell viability assay [43]. Briefly, cell viability was determined on the basis of changes in cellular ATP levels using the CellTiter-Glo Luminescent Cell Viability Assay kit (Promega, Madison, WI) according to the manufacturer’s instructions. Following treatment with test agents, cell suspension was incubated with ATP assay buffer at room temperature for 10 min. Luminescence was then measured in quadruplicate using a SpectraMax Paradigm (Molecular Devices Co., Sunnyvale, CA, USA) plate reader.

### 3.4. Assessment of Apoptosis by Annexin-V and Propidium Iodide Staining

HL60 cells were washed with PBS and were incubated with 50 μg/mL annexin-V-APC and 10 μg/mL propidium iodide in binding buffer at room temperature in the dark, for 15 min, and analyzed in a Gallios flow cytometer (Beckman Coulter, Miami, FL, USA), as described previously [14,15]. Ten thousand events were acquired for each sample and the data were analyzed using the Kaluza Analysis Software version 2.1.1 (Beckman Coulter). Annexin-V-positive/PI-negative cells were considered to be early apoptotic, cells positive for both annexin-V and PI to be late apoptotic, and annexin-V-negative but PI positive cells to be necrotic. The total apoptosis was calculated as the sum of the percentages of early and late apoptotic cells.

### 3.5. Preparation of Whole Cell Lysates and Western Blot Analysis

HL60 and U937 cells (2 × 10^6^) were plated in 6-well plates, incubated with NUP for 24 h and lysed in a buffer containing 1% (*v*/*v*) Triton X-100 at 4 °C, as described previously [14,15]. Equal amounts of protein (30 μg) were separated by SDS-PAGE and electroblotted into nitrocellulose membrane. The membranes were blocked with 5% nonfat milk in Tris-buffered saline containing 0.5% Tween 20 (TBST) for 2 h and incubated with the primary antibodies overnight at 4 °C. The following primary antibodies were used: caspase-3 (Santa Cruz Biotechnology; cat. # sc-7148, 1:500), cleaved caspase-3 (Cell Signaling Technology; cat. # 9661; 1:500), caspase-8 (Cell Signaling Technology; cat. # 9746; 1:500), caspase-9 (Cell Signaling Technology; cat. # 9502; 1:500), poly(ADP-ribose) polymerase (PARP) (Enzo Life Sciences; cat. # BML-SA253, 1:2000). Blots were washed and incubated with horse-radish peroxidase-conjugated anti-rabbit (ImmunoResearch Laboratories, West Grove, PA, USA) or anti-mouse (GE Healthcare, Pittsburg, PA, USA) secondary antibodies. The protein bands were visualized using the Western Lightning™ Chemiluminescence Reagent Plus (PerkinElmer Life Sciences, Inc., Boston, MA, USA).

### 3.6. Determination of Intracellular Levels of Reactive Oxygen Species (ROS)

The intracellular ROS levels were determined using the oxidation-sensitive fluorescent indicator DCFH-DA. Intracellular ROS oxidize this probe to a highly fluorescent compound 2′,7′-dichlorofluorescein (DCF). Following different treatments, cells (5  ×  10^5^) were harvested, washed with HBSS containing 10 mM HEPES (pH = 7.4) and loaded with 5 μM DCFH-DA, for 15 min at 37 °C in a shaking water bath [11]. Cells were washed and resuspended in HBSS followed by flow cytometric analysis. For a positive control, untreated cells loaded with DCFH-DA were washed and incubated with 0.5 mM H_2_O_2_ for 30 min under the same conditions. The fluorescence intensity was measured in a Gallios flow cytometer. For each analysis, 10,000 events were recorded. The data were analyzed using Kaluza Analysis Software version 2.1.1.

### 3.7. Determination of Intracellular Glutathione (GSH) Levels

GSH levels were determined in HL60 cells using the Intracellular GSH Assay kit. Cells (5  ×  10^5^) were harvested, washed with HBSS containing 10 mM HEPES (pH = 7.4), and loaded with 5 µM ThioBright Green for 30 min in accordance with the manufacturer’s recommended protocol. Cells were then washed and resuspended with HBSS containing 10 mM HEPES. The fluorescence intensity was measured in a Gallios flow cytometer. For each analysis, 10,000 events were recorded. The data were analyzed using Kaluza Analysis software version 2.1.1.

### 3.8. Measurements of Steady-State Cytosolic Ca^2+^ Levels

HL60 cells (3–4 × 10^5^/mL) were treated with test compounds for 2 h and 4 h, washed, and resuspended in Ca^2+^-supplemented (2 mM) Ringer’s solution containing 2.35 μM Fluo-3/AM, as described previously [14]. Cells were then incubated in the dark for 30 min at room temperature, washed, and resuspended in Ca^2+^-free Ringer’s solution. The fluorescence intensity was measured in a Gallios flow cytometer (Beckman Coulter, Miami, FL, USA). For each analysis, 10,000 events were recorded. Data were processed using Kaluza Analysis Software version 2.1.1 (Beckman Coulter).

### 3.9. Statistical Analysis

All experiments were conducted at least three times. The data are presented as means ± SD. The significance of the differences between different experimental groups was estimated by unpaired, two-tailed Student’s *t*-test. *p* < 0.05 was considered statistically significant. All statistical analyses were performed with the GraphPad Prism 7.0 program (GraphPad Software, San Diego, CA, USA).

## 4. Conclusions

In conclusion, our study demonstrates that both the thioalkaloid-enriched NUP and its purified component DTNB are capable of inducing massive apoptotic cell death of AML cells, but not of normal human white blood cells. NUP-induced apoptosis was found to be mediated, at least in part, by the induction of oxidative stress and Ca^2+^_cyt_ accumulation, whereas the proapoptotic effect of DTBN appears to mainly involve ROS- and Ca^2+^-independent cellular regulatory pathways. Further studies are required to elucidate the molecular mechanisms underlying the observed antileukemic actions of NUP and DTBN.

*N. lutea* extracts and purified nupharidine compounds have been shown to exert a wide array of biological effects. They have potentially useful applications in medicine, including cancer, that need to be further explored and validated in translational studies to determine their therapeutic windows. Knowing more about the mechanism(s) of action of *N. lutea* constituents, including nupharidines, may lead to the synthesis of their molecular analogs with improved anticancer activity for the use as single agents or in combination with other treatment modalities.

## Figures and Tables

**Table 1:** Comparison of the cytotoxic potency of NUP in KG-1a, HL60, and U937 human AML cell lines at different time points (IC_50_ values).

Cell Line	24 h	48 h	72 h
KG-1a	4.06 ± 0.33	1.21 ± 0.15	0.90 ± 0.58
HL60	2.40 ± 0.14	1.03 ± 0.14	1.09 ± 0.13
U937	1.65 ± 0.15	0.97 ± 0.18	0.89 ± 0.05 ^1^

^1^ The IC_50_ values (µg/mL) were calculated by nonlinear regression analysis of the dose-response curves for the reduction in the number of viable cells, as shown in Supplementary Figure S1 Data are the means ± SD (*n* = 3).

## Data Availability

Data is contained in the article and supplementary Materials.

## Supplementary Materials

The following are available online at https://www.mdpi.com/article/10.3390/ph15040410/s1, Figure S1: Chemical structures of several dimeric sesquiterpene thioalkaloids found in NUP.
