# Supplementary material for: Cytotoxicity of Thioalkaloid-Enriched Nuphar lutea Extract and Purified 6,6′-Dihydroxythiobinupharidine in Acute Myeloid Leukemia Cells: The Role of Oxidative Stress and Intracellular Calcium
Source: Pharmaceuticals (Basel). 2022 Mar 28;15(4):410. doi: 10.3390/ph15040410 (PMC9032197; doi:10.3390/ph15040410)
Supplement: Supplementary file 1 [file pharmaceuticals-15-00410-s001.zip › pharmaceuticals-1560203-supplementary.pdf]

Cytotoxicity of thioalkaloid-enriched *Nuphar lutea* extract and purified 6,6′-dihydroxythiobinupharidine in acute myeloid leukemia cells: The role of oxidative stress and intracellular calcium

Suchismita Muduli [1], Avi Golan-Goldhirsh [2], Jacob Gopas [3,4] and Michael Danilenko [1]*

[1] Department of Clinical Biochemistry & Pharmacology, Faculty of Health Sciences, Ben-Gurion University of the Negev, Beer Sheva, Israel; muduli@post.bgu.ac.il (S.M.), misha@bgu.ac.il (M.D.)

[2] The Jacob Blaustein Institutes for Desert Research (BIDR), French Associates Institute for Agriculture and Biotechnology of Drylands, Ben-Gurion University of the Negev, Sede Boqer Campus, Israel; avigolan@bgu.ac.il

[3] Department of Microbiology, Immunology & Genetics; Faculty of Health Sciences, Ben-Gurion University of the Negev, Beer Sheva, Israel; jacob@bgu.ac.il

[4] Department of Oncology, Soroka University Medical Center, Beer Sheva, Israel; jacob@bgu.ac.il

* Correspondence: misha@bgu.ac.il; Tel: +972-8-647-9979

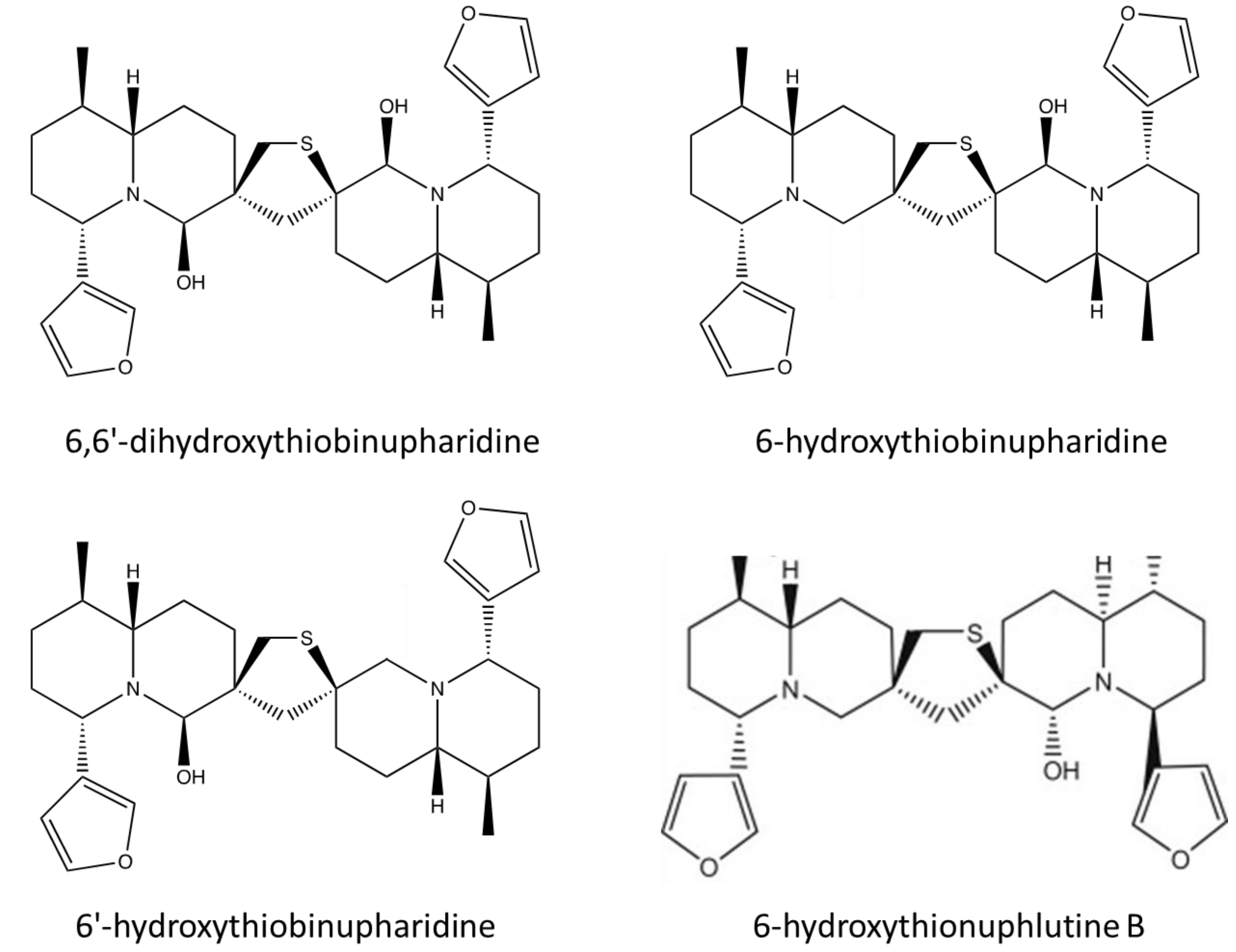

Supplementary Figure S1. Chemical structures of several dimeric sesquiterpene thioalkaloids found in NUP.
